# An open label pilot study to evaluate the efficacy of Spanish black radish on the induction of phase I and phase II enzymes in healthy male subjects

**DOI:** 10.1186/1472-6882-14-475

**Published:** 2014-12-09

**Authors:** Malkanthi Evans, Elaine Paterson, David M Barnes

**Affiliations:** KGK Synergize, Inc., 255 Queens Ave., London, ON N6A 5R8 Canada; Standard Process, Inc., 1200 Royal Lee Drive, Palmyra, WI 53156 USA

**Keywords:** Spanish black radish, Detoxification, Hepatic function

## Abstract

**Background:**

Humans are exposed to toxins which accumulate in the body, and are detoxified primarily in the liver. Studies have shown that cruciferous vegetables (such as radishes) may be beneficial to health by aiding detoxification of toxins in the liver.

**Methods:**

This single-centre, open-label, pilot study investigated the effect of a dietary supplement containing Spanish Black Radish on hepatic function in healthy males by monitoring the profiles of plasma and urine acetaminophen metabolites and serum hormone concentrations at baseline and after 4 weeks of supplementation. A paired t-test was used to compare pre- and post-treatment of plasma and urine acetaminophen metabolite profiles, serum hormone concentrations and safety end points.

**Results:**

Area under the curve (AUC) from 0 to 8 hours for the acetaminophen glucuronide metabolite and unchanged acetaminophen in plasma decreased from baseline to week 4 by 9% (*P* = 0.004) and 40% (*P* = 0.010), respectively. The AUC from 0 to 8 hours for acetaminophen sulfate and mercapturate metabolites in the urine increased by 11% (*P* = 0.010) and 37% (*P =* 0.024), respectively, from baseline to week 4. The AUC from 0 to 8 hours of serum estradiol-17β decreased by 10% from baseline to week 4 (*P* = 0.005). All measures of clinical safety remained within acceptable laboratory ranges, however a significant reduction in plasma γ-glutamyl transferase levels was noted after 4 weeks of Spanish Black Radish treatment (*P = 0.002*).

**Conclusions:**

These changes in metabolite and hormone levels indicate that Spanish Black Radish supplements have a positive influence on the detoxification of acetaminophen suggesting up-regulation of phase I and phase II liver enzymes. This study was sponsored by Standard Process Inc.

**Trial registration:**

ClinicalTrials.gov registration number NCT02137590 (Date of registration: May 12, 2014)

## Background

Humans are exposed to numerous environmental, endogenous and man-made toxins. Many of these compounds are lipophilic and accumulate in the body [1], requiring detoxification to avoid negative impacts on metabolism. The role of diet in modifying toxin metabolism has been suggested, but whether this action might influence health status remains uncertain. Cruciferous vegetables have been investigated for their ability to induce detoxification enzymes when consumed both as a fresh food and as a supplement [1–3].

Detoxification takes place primarily in the liver [4], which performs the crucial task of converting lipophilic toxins to more hydrophilic metabolites that can be eliminated from the body via urine [1]. The detoxification process happens in two phases [5]. During the phase I detoxification process, a toxic compound is made more polar through enzymatic action in a process called biotransformation, a selective process by which drugs, hormones and toxins are deactivated by enzyme catalysis [5]. Cytochrome P450 (CYP) is a family of more than 25 enzymes involved in the critical phase I system whose actions generate free radicals and reactive oxygen species. Phase I reactions may also expose functional groups in drugs and toxic compounds for more effective phase II detoxification. In the phase II detoxification process, free radicals and reactive oxygen species are deactivated by conjugation. Balance between phase I and phase II reactions is important to prevent accumulation of toxic intermediates.

Metabolism of acetaminophen by the liver occurs through three detoxification pathways: glucuronidation, sulfation and glutathione (GSH) conjugation. This makes acetaminophen metabolism a good model to study the detoxification pathways involved in protection against exposure to a wide range of exogenous and endogenous toxins. Acetaminophen metabolism occurs in the liver predominantly by sulfation and glucoronidation, both phase II conjugation reactions. Acetaminophen glucuronide and acetaminophen sulfate are non-toxic and excreted in urine. A small proportion (5-10%) of the acetaminophen is oxidized through phase I reactions by CYP enzymes. The oxidation of acetaminophen leads to the formation of N-acetyl-p-benzoquinone imine (NAPQI). Unconjugated NAPQI is a toxic compound that requires further metabolism with GSH to form acetaminophen mercapturate and acetaminophen cysteine, two non-toxic metabolites [6, 7].

Two key components in toxin metabolism are enzymatic activity and availability of metabolic cofactors. Cofactors like GSH are necessary in the metabolism of NAPQI to form non-toxic conjugates, such as mercapturic acid, which are targeted for excretion. When GSH is deficient, NAPQI binds to sulfhydryl proteins in the liver, leading to lipid peroxidation and subsequent hepatic cell damage. Impaired metabolic function of the liver can be assessed indirectly by acetaminophen conjugates.

Spanish Black Radish (SBR, *Raphinoussativus* L. Var. *niger*) is a cruciferous vegetable which contains high concentrations of glucosinolates, as well as sulfates and cysteine-rich proteins which are precursors for GSH synthesis [8, 9]. *In vitro* and *in vivo* models have demonstrated that SBR induces both phase I and II enzymes [8, 10]. A diet consisting of 20% freeze-dried radish has been shown *in vivo* to stimulate CYP isoenzymes, CYP 1A1 and CYP 1A2, phase I detoxification enzymes, and glutathione S-transferase, quinone reductase and microsomal epoxide hydrolase, phase II detoxification enzymes [10] *in vivo*. In addition, recent evidence showed that a 20% radish diet protected mice following exposure to the model carcinogen, 7,12-dimethylbenz(a)anthracene (DMBA) through both phase I and II enzymes in DMBA clearance [11]. Ingestion of radishes induces detoxification enzymes in mice [10] and although many human studies investigating the effects of fresh crucifers [12–14] have been published, few studies have investigated the supplement form of crucifers [15, 16].

Therefore, the purpose of this study was to investigate the effect of an SBR supplement on hepatic function by examining acetaminophen and steroid hormone metabolism dependent on phase I and phase II enzyme activities. Phase I and II enzyme activity was evaluated via various serum hormones, as well as plasma and urine acetaminophen metabolite concentrations following administration of 1000 mg acetaminophen prior to and after a 28 day supplementation period. The study also evaluated the safety of SBR by monitoring complete blood count, kidney function (creatinine), liver function (aspartate aminotransferase [AST], alanine aminotransferase [ALT], gamma-glutamyltransferase [GGT]), electrolytes (sodium, potassium, and chloride), vital signs (heart rate and blood pressure) and adverse events.

## Methods

### Study participants

This single-center, open-label, pilot study was conducted at KGK Synergize Inc, in London, ON, Canada, from September 2011 to November 2011. The study was reviewed by the Natural Health Products Directorate (NHPD) (Health Canada, Ottawa, ON). Notice of authorization was granted on August 16, 2011 by the NHPD and ethics approval was received from Institutional Review Board Services (Aurora, ON) on September 02, 2011. The study was conducted in accordance with the ethical principles that have their origins in the Declaration of Helsinki and its subsequent amendments. Written informed consent was obtained from subjects prior to any study procedures.

Study participants were recruited from a research subject database and by newspaper advertisements. Twenty healthy male subjects, age 25–35 years, with a body mass index (BMI) between 18 and 25 kg/m^2^ were enrolled after screening and passing eligibility criteria. Participants were required to avoid the consumption of cruciferous vegetables for 14 days prior to and during the study treatment period. Participants were excluded if they had: any chronic diseases or medical conditions, including gall bladder disorders and/or bowel obstruction or were on prescription or over the counter medications for the treatment of any acute or chronic conditions. Subjects who were taking muscle building supplements, products containing ingredients derived from the Cruciferae (or Brassicaceae) plant family, or natural health products other than vitamins or minerals within 14 days of baseline were ineligible to participate. Subjects with clinically-significant abnormal laboratory values, allergies or sensitivities to test product ingredients, Cruciferae plant family (including mustard, cabbage, radish), acetaminophen, or the food and beverages provided during the study, or who had used acetaminophen within 48 hrs of baseline were also excluded.

### Study design and investigational product

At screening, participant’s medical history and concomitant therapies were reviewed and heart rate and blood pressure measured and BMI calculated. Blood samples were obtained to determine complete blood count, electrolytes (sodium, potassium and chloride), creatinine, aspartate aminotransferase, alanine aminotransferase, γ-glutamyl transferase and bilirubin. Participants were instructed to avoid cruciferous vegetables for 14 days prior to enrollment and for the duration of the study. Subjects were provided with a list of meal items, devoid of cruciferous vegetables, for the two 8-hour test days and selected their food preferences. Participants were instructed to avoid alcohol consumption for 24 hours and to fast for 12 hours prior to their baseline and their final visit on Day 28.

Participants returned to the clinic for their 8-hour in-clinic baseline visit (week 0) at which time their heart rate and blood pressure were measured and BMI calculated. Acetaminophen was administered at a dose of 1000 mg (Tylenol Extra Strength 500 mg caplets DIN 00723908, McNeil Consumer Healthcare, Markham, ON). Blood and urine samples were collected at pre-acetaminophen dose (0 hours) and at 2, 4, 6 and 8 hours post-acetaminophen dose for the analysis of plasma acetaminophen metabolites (glucuronide, sulfate, and NAPQI-GSH), urine metabolites (glucuronide, mercapturate and sulfate), unchanged acetaminophen concentrations in plasma and urine, serum total testosterone, free testosterone and estradiol-17β. Meals were provided after the pre-dose and 4-hour blood collection. After the 8-hour test period, participants received 3-day food records and paper diaries for recording their study product use, changes in concomitant therapies, and any side effects/changes in health conditions.

The investigational product, SBR (One tablet: 370 mg of SBR, 15.33 mg camu camu (*Myrciaria dubia*), 18.61 mg acerola (*Malpighia emarginata)*, honey, manoic root (tapioca) and calcium stearate; Standard Process Inc., Palmyra, WI), was dispensed to the participants during their baseline visit and at every week thereafter for up to 3 weeks. Participants were instructed to begin consuming 6 tablets daily (2 tablets at breakfast, lunch and supper) the day following their baseline visit. Participants returned to the clinic on day 7, 14, 21 and 28 post-baseline at which time the remaining product and packaging was returned to determine compliance with the treatment regimen.

Participants returned to the clinic in a fasting state at 28 days post-baseline (week 4) for an 8-hour in-clinic test at which time heart rate and blood pressure were measured and BMI was calculated. Participants were administered 1000 mg of acetaminophen (2 Tylenol Extra Strength 500 mg caplets, McNeil Consumer Healthcare). Blood and urine samples were collected pre-acetaminophen dosage and at 2, 4, 6 and 8 hours post-acetaminophen dosage for analysis of plasma and urine acetaminophen metabolites (glucuronide, sulfate and NAPQI-GSH), urine metabolites (glucuronide, sulfate and mercapturate), unchanged acetaminophen concentrations in plasma and urine, and serum total testosterone, free testosterone and estradiol-17β. Meals were provided after the pre-dose and the 4-hour blood collection. Subjects were required to consume the same meal items on each of the 8-hour test days.

### Food record and diary

A three-day food record was dispensed at screening and at each subsequent visit. Participants were instructed to complete it weekly on two weekdays and one weekend day, for the two weeks prior to the baseline visit and every week thereafter up to day 28. Food records were used to ensure that participants were compliant to the food regimen and did not consume cruciferous vegetables as instructed. A paper diary was dispensed to subjects at baseline and on Day 7, 14 and 21 for subjects to record product use, changes in concomitant therapies, and any side-effects/changes in health conditions. The diaries were reviewed weekly.

### Treatment compliance

Compliance was assessed by counting the returned tablets at each visit. The compliance rate was calculated by dividing the number of tablets consumed divided by the number expected to have been taken multiplied by 100. Participants were counseled on product use at a compliance of <80% or >120% at any visit. Participants with a compliance rate of <75% or >130%, for two consecutive visits, were considered non-compliant and withdrawn from the study.

### Analysis of plasma and urine acetaminophen metabolites

#### Sample collection and preparation

Whole blood was collected in 6 ml heparin tubes at 0 (pre-acetaminophen dose), 2, 4, 6 and 8 hours post-acetaminophen dose during baseline and on Day 28. The plasma was separated and aliquoted into 1.0 mL volume samples. These samples were stored below -40°C until high-performance lipid chromatography (HPLC) analysis was performed. Plasma samples were prepared for HPLC analysis by adding 25 μl of 30% w/v aqueous perchloric acid (311413, Sigma-Aldrich, Toronto, Canada) to 250 μl of plasma in a 1.5 ml Eppendorf tube and then vortexed for 30 seconds and centrifuged at 1800 g for 10 min and 20 μl of clear supernatant was injected into the HPLC system.

At baseline and week 4, urine was collected in separate containers at 2 hour intervals between 0 and 8 hours, aliquoted into 1.0 mL volumes and stored below -40°C until HPLC analysis. Urine samples were prepared for HPLC analysis by adding 750 μl of Milli Q water to 250 μl of urine and vortexed. The preparation was centrifuged at 1800 g for 10 min and 20 μl of the preparation was injected into the HPLC system.

#### Preparation of standards for plasma analysis

Paracetamol (50 μg, A5000, Sigma-Aldrich, Toronto, Canada), paracetamol glucuronide (50 μg, A4438, Sigma-Aldrich, Toronto, Canada), or paracetamol sulfate (50 μg, A161230, Toronto Research Chemical Inc., Toronto, Canada) was added to 1 ml of Milli Q water/methanol mixture (50:50 v/v). NAPQI (50 μg, A170000, Toronto Research Chemical) was added to 1 ml acetonitrile. Eight standard solutions were prepared in plasma devoid of paracetamol (blank) at the following concentrations: 25 μg/ml, 12.5 μg/ml, 6.25 μg/ml, 3.125 μg/ml, 1.56 μg/ml, 0.78 μg/ml and 0.39 μg/ml by serial dilution.

#### Preparation of standards for urine analysis

Paracetamol (5000 μg, A5000, Sigma-Aldrich), paracetamol glucuronide (5000 μg, A4438, Sigma-Aldrich), paracetamol sulfate (5000 μg, A161230, Toronto Research Chemical) or paracetamol mercapturate (5000 μg, A172100, Toronto Research Chemical) were added to 1 ml of a Milli Q water/methanol mixture (50:50 v/v). Serial dilutions of each stock solution were prepared in urine devoid of paracetamol (blank) to give the following concentrations: 2500 μg/ml, 1250 μg/ml, 625 μg/ml, 312.5 μg/ml, 156.25 μg/ml, 78.15 μg/ml, 39.0625 μg/ml, 19.531 μg/ml, 9.766 μg/ml and 4.883 μg/ml by serial dilution.

#### Chromatographic conditions

The chromatography system comprised of a Varian Solvent Delivery module 210, injector Varian Auto Sampler model 410, Varian 335 Photo Diode Array Detector for detection and MS workstation software 6.4.1 for data acquisition. Reverse phase isocratic HPLC analysis was performed with a C18 column with particle size 4 μm (4.6x150 mm). The flow rate was 0.65-0.75 ml/minute and the detection was at 240–245 nm. A 0.1 M sodium phosphate buffer containing acetonitrile at a ratio of 10:90 (v/v) was used as the mobile phase for plasma analysis. A 1 M sodium phosphate buffer containing acetonitrile at a ratio of 5:95 (v/v) was used as mobile phase for urine analysis.

### Analysis of serum hormones

Two 5 ml serum separating tubes of blood were collected for serum total/free testosterone and estradiol-17β at each time-point (0, 2, 4, 6, 8 h). The samples were allowed to clot at room temperature for 30 minutes and then centrifuged for 10 minutes at 3200 rpm at 25°C and shipped refrigerated to a Central Laboratory for analysis (LifeLabs Medical Laboratory Services, London, ON, Canada).

### Statistical analysis

A paired t-test was used to compare pre- and post-treatment efficacy measurements of serum and urine acetaminophen metabolite levels, and for plasma testosterone and estradiol-17β. Statistical comparisons of maximum concentrations (C_max_) and area under the concentration time curve (AUC) were performed on log transformed data. A paired t-test was used to compare pre- and post-treatment measurements of safety end points: biometrics, vital signs, hematology and blood clinical chemistry parameters. SAS Version 9.1 was used to perform the statistical analysis, with probability values ≤0.05 considered statistically significant.

## Results

### Recruitment and compliance

**Figure 1:**
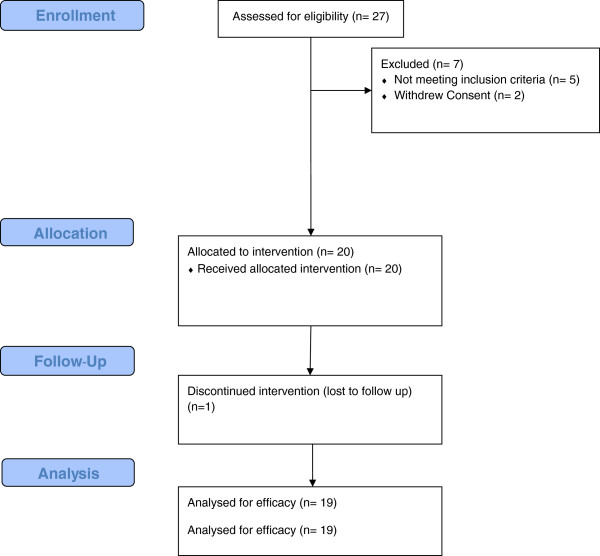
**Disposition of study subjects.**

A total of 27 subjects were screened and 20 eligible subjects were enrolled (Figure 1). Nineteen participants completed the study and were included in the analysis of efficacy and safety. One subject was lost to follow up after the baseline visit and had no post-baseline data available for analysis. The enrolled participants had a mean age of 28.9 years and a mean BMI of 23.6 kg/m^2^ (Table 1). Eighty-five percent of the subjects were of Western European origin. Mean compliance with the treatment regimen was >90% throughout the investigational period.

**Table 1:** **Demographics and characteristics of subjects in the study**

	All subjects at screening (N = 20)	All subjects completing the study (N = 19*)
Age (years)	28.85 ± 3.10	28.84 ± 3.18
Weight (kg)	73.18 ± 6.91	73.55 ± 6.90
BMI (kg/m^2^)	23.56 ± 1.50	23.49 ± 1.51
Mean systolic blood pressure (mmHg)	112.95 ± 6.90	113.21 ± 6.99
Mean diastolic blood Pressure (mmHg)	71.50 ± 7.78	71.47 ± 8.00
Mean heart rate (bpm)	68.05 ± 8.56	67.84 ± 8.74

Values are presented as Mean ± SD. BMI indicates body mass index.

*One subject was lost to follow up.

### Acetaminophen metabolites in plasma

**Figure 2:**
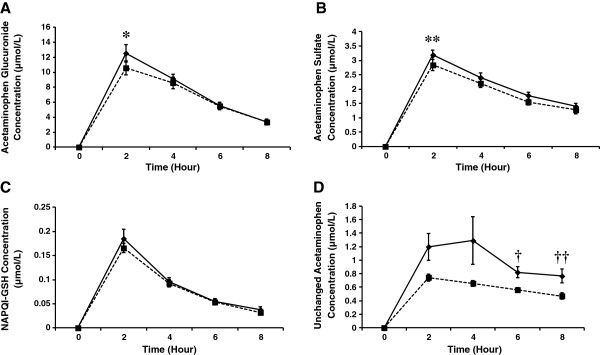
**The plasma concentration of acetaminophen metabolites at baseline and 4 weeks.** Plasma profiles of acetaminophen glucuronide **(A)**, acetaminophen sulfate **(B)**, NAPQI-GSH **(C)** and unchanged acetaminophen **(D)** of pre-acetaminophen dose (0 h) and 2, 4, 6 and 8 h following administration of 1000 mg acetaminophen to subjects (N = 19) at week 0 (♦) and after 4 weeks of SBR supplementation (_▀,_ broken line) (mean ± SEM). *P < 0.001, **P = 0.046, †*P* = 0.019, and ††*P* = 0.031, between week 0 and 4 weeks of SBR supplementation.

Plasma profiles were similar for acetaminophen glucuronide, sulfate, and NAPQI-GSH at week 0 (baseline) and after 4 weeks of supplementation with SBR. Glucuronide and sulfate concentrations were similar at pre-acetaminophen dose but decreased significantly at 2 h post-acetaminophen dose (*P* < 0.001, Figure 2A and *P* = 0.046, Figure 2B, respectively) after a 4 week supplementation with SBR.

NAPQI-GSH concentrations were similar at all time points at week 0 and after the 4-week supplementation with SBR (Figure 2C). The plasma profile of unchanged acetaminophen after 4 weeks of supplementation with SBR showed lower concentrations than its profile at week 0. Plasma concentrations at 6 h and 8 h were significantly lower (*P* = 0.019 and *P* = 0.031, respectively) after the 4-week supplementation period (Figure 2D). There was a significant decrease in the C_max_ of plasma glucuronide and unchanged acetaminophen (*P* < 0.001 and *P* = 0.022, respectively). Plasma glucuronide and unchanged acetaminophen AUC from 0 to 8 hours were significantly decreased after the 4-week SBR supplementation compared to week 0 (*P* = 0.004 and *P* = 0.010, respectively) (Table 2). By the end of the 4-week supplementation period, plasma levels of acetaminophen glucuronide, sulfate, NAPQI-GSH and unchanged acetaminophen AUC_0-8h_ demonstrated 9%, 10%, 9% and 40% decreases from week 0, respectively (Table 2).

**Table 2:** **Plasma profile of acetaminophen metabolites at baseline and 4 weeks**

Plasma acetaminophen metabolite	Week 0	Week 4	Difference from week 0 to week 4	***P***Value ^a^
**Acetaminophen glucuronide**				
C_max_(μmol/L)	12.59 ± 4.12	10.82 ± 3.091	-1.77 ± 1.47	<0.001
T_max_(h)	2.13 ± 0.46	2.55 ± 1.13	0.42 ± 1.26	0.166
AUC_(0–8 hour)_ (μmol. h/L)	58.05 ± 14.48	52.83 ± 14.09	-5.22 ± 6.32	<0.004
**Acetaminophen sulfate**				
C_max_(μmol/L)	3.20 ± 0.69	2.90 ± 0.82	-0.30 ± 0.69	0.085
T_max_(h)	2.03 ± 0.01	2.23 ± 0.63	0.21 ± 0.63	0.169
AUC_(0–8 hour)_ (μmol. h/L)	8.78 ± 4.073	9.64 ± 4.02	-1.69 ± 4.07	0.126
**NAPQI-GSH**				
C_max_(μmol/L)	0.18 ± 0.09	0.18 ± 0.04	-0.02 ± 0.07	0.200
T_max_(h)	2.03 ± 0.01	2.02 ± 0.01	-0.00 ± 0.02	0.682
AUC_(0–8 hour)_ (μmol. h/L)	0.72 ± 0.31	0.66 ± 0.22	-0.07 ± 0.22	0.336
**Unchanged acetaminophen**				
C_max_(μmol/L)	1.59 ± 1.65	0.79 ± 0.20	-0.79 ± 1.72	0.022
T_max_(h)	2.87 ± 1.38	3.08 ± 1.68	0.21 ± 2.30	0.696
AUC_(0–8 hour)_ (μmol. h/L)	4.10 ± 2.25	2.45 ± 0.66	-1.65 ± 2.65	0.010

C_max_, T_max_ and AUC_0-8h_ of plasma acetaminophen metabolites following administration of 1000 mg acetaminophen to participants at week 0 and after 4 weeks of SBR supplementation.

Values are presented as Mean ± SD.

^a^Between group comparisons were made using *t*-test. Statistical comparisons for C_max_ and AUC_(0–8 hour)_ were conducted using log-transformed data.

C_max_ = maximum concentration; T_max_ = time of maximum concentration; AUC = area under the plasma concentration time curve.

### Acetaminophen metabolites in urine

**Figure 3:**
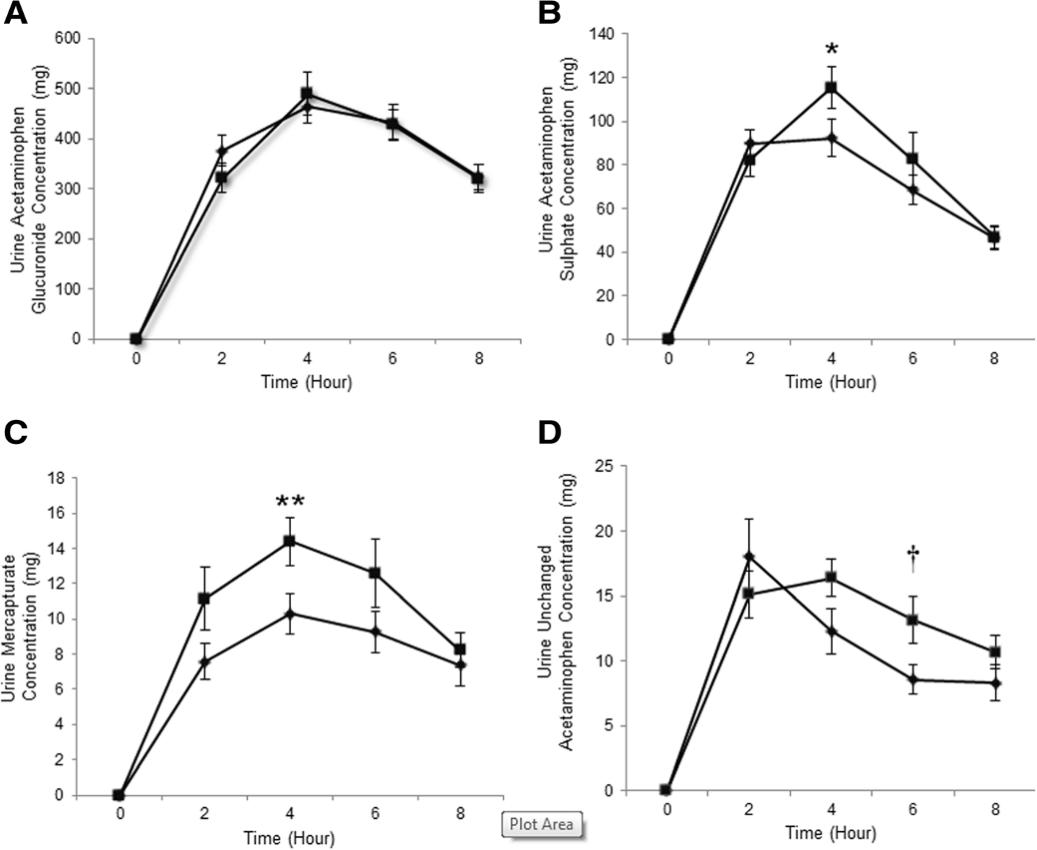
**The urine concentration of acetaminophen metabolites at baseline and 4 weeks.** Urine profiles of acetaminophen glucuronide **(A)**, acetaminophen sulfate **(B)**, mercapturate **(C)** and unchanged acetaminophen **(D)** of pre-acetaminophen dose (0 h) and 2, 4, 6 and 8 h following administration of 1000 mg acetaminophen to subjects (n = 19) at week 0 (♦) and after 4 weeks of SBR supplementation (_▀,_ broken line) (mean ± SEM). *P = 0.021, **P = 0.019 and †*P* = 0.035, between week 0 and after 4 weeks of SBR supplementation.

The patterns of excretion for urine acetaminophen glucuronide, sulfate, and mercapturate were similar at week 0 and after 4 weeks of supplementation with SBR. There were no significant differences in urine acetaminophen glucuronide concentration at week 0 and after the 4-week supplementation with SBR for any of the time-points measured (Figure 3A). There were no significant differences in glucuronide C_max_, T_max_ or AUC_0-8h_ between week 0 and after the 4-week SBR supplementation period (Table 3).

**Table 3:** **Urine profile of acetaminophen metabolites at baseline and 4 weeks**

Urine acetaminophen metabolites	Week 0	Week 4	Difference from week 0 to week 4	***P***Value ^a^
**Acetaminophen glucuronide**				
C_max_(mg)	534.34 ± 100.71	553.91 ± 151.27	19.57 ± 124.28	0.661
T_max_(h)	4.95 ± 1.54	5.26 ± 1.52	0.32 ± 1.67	0.420
AUC_(0–8 hour)_ (mg. h)	2842.35 ± 654.09	2803.66 ± 655.02	-38.69 ± 342.45	0.675
**Acetaminophen sulfate**				
C_max_(mg)	109.65 ± 27.66	128.72 ± 48.28	19.08 ± 38.37	0.030
T_max_(h)	3.37 ± 1.34	4.21 ± 1.47	0.84 ± 1.92	0.072
AUC_(0–8 hour)_ (mg. h)	541.92 ± 117.29	603.26 ± 197.79	61.34 ± 166.19	0.010
**Acetaminophen mercapturate**				
C_max_(mg)	12.41 ± 4.81	17.23 ± 8.27	4.82 ± 9.72	0.027
T_max_(h)	4.84 ± 1.80	4.21 ± 1.62	-0.63 ± 2.50	0.285
AUC_(0–8 hour)_ (mg. h)	61.56 ± 25.66	84.24 ± 35.78	22.68 ± 44.43	0.024
**Unchanged acetaminophen**				
C_max_(mg)	18.95 ± 11.01	20.91 ± 7.42	1.96 ± 10.97	0.151
T_max_(h)	3.05 ± 1.81	3.89 ± 1.94	0.84 ± 2.77	0.202
AUC_(0–8 hour)_ (mg.h)	84.02 ± 45.36	99.33 ± 31.98	15.31 ± 45.65	0.060

C_max_, T_max_ and AUC_0-8h_ of urine acetaminophen metabolites following administration of 1000 mg acetaminophen to participants at week 0 and after 4 weeks of SBR supplementation.

Values are presented as Mean ± SD.

^a^Between group comparisons were made using *t*-test. Statistical comparisons for C_max_ and AUC_(0-8hour)_ were conducted using log-transformed data.

C_max_ = maximum concentration; T_max_ = time of maximum concentration; AUC = area under the urine concentration time curve.

Urine sulfate concentrations were significantly higher 4 h post-acetaminophen dose after 4 weeks of SBR supplementation compared to week 0 (*P* = 0.021) (Figure 3B). There was a significant increase in urine sulfate C_max_ (*P* = 0.030) and AUC_0-8h_ (*P* = 0.010) at week 4 compared to week 0 (Table 3). By the end of the 4-week supplementation with SBR, urine levels of acetaminophen sulfate increased by 11%.

Urine acetaminophen mercapturate concentrations were higher at all time points, however, significance was only reached at 4 h post-acetaminophen dose (*P* = 0.019) after 4 weeks of SBR supplementation when compared to week 0 (Figure 3C). There was a significant increase in urine mercapturate C_max_ (*P* = 0.027) and AUC_(0-8h)_ (*P* = 0.024) after the 4-week supplementation with SBR compared to week 0 (Table 3). By the end of the 4-week supplementation, urine levels of mercapturate metabolite had increased by 37%.

The pattern for urinary unchanged acetaminophen was similar at week 0 and after 4 weeks of SBR supplementation, however concentrations at week 4 reached a peak later than at week 0 (4 h vs. 2 h). Concentrations of unchanged acetaminophen were higher at 4 h, 6 h, and 8 h post-acetaminophen dose after the 4-weeks of SBR supplementation, with statistical significance (*P* = 0.035) reached only at 6 h post-acetaminophen dose (Figure 3D). A trend toward a significant increase was seen for urine unchanged acetaminophen AUC_0-8h_ at week 4 compared to week 0 (*P* = 0.060) (Table 3). By the end of the 4-week supplementation with SBR, urine levels of unchanged acetaminophen had increased by 18%.

### Hormone levels in serum

**Figure 4:**
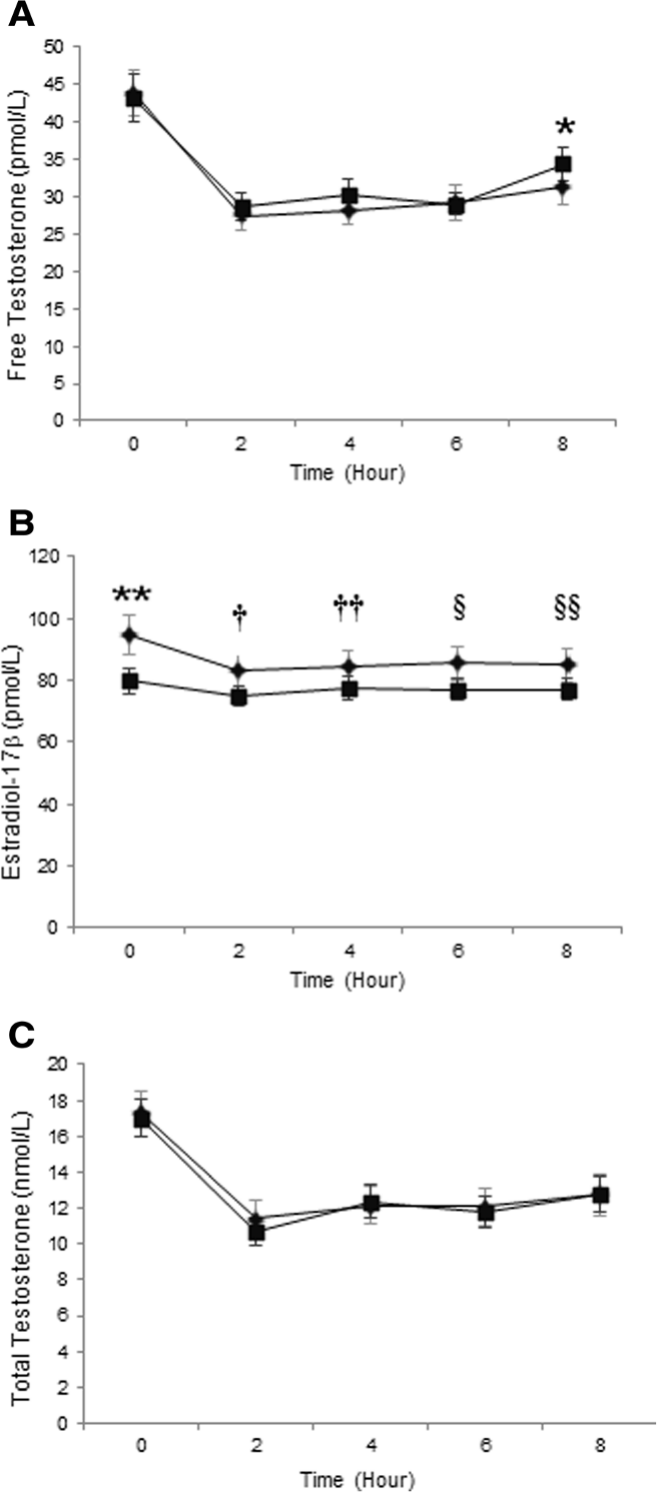
**The serum concentration of hormones at baseline and 4 weeks.** The serum profiles of free testosterone **(A)**, estradiol-17 β **(B)** and total testosterone **(C)** at pre-acetaminophen dose (0 h) and 2, 4, 6 and 8 h following administration of 1000 mg acetaminophen to subjects (n = 19) at week 0 (♦) and after 4 weeks of SBR supplementation (_▀,_ broken line) (mean ± SEM). *P = 0.022, **P = 0.005*,* †*P* = 0.007, ††*P* = 0.040, §*P* = 0.021 and §§*P* = 0.046, between baseline and after 4 weeks of SBR supplementation.

Post-acetaminophen dose serum concentration time-profile of total testosterone, free testosterone and estradiol-17β followed a similar pattern at week 0 and after 4 weeks of supplementation with SBR (Figure 4). There was a significant increase in serum free testosterone at 8 h post-acetaminophen dose after 4 weeks of supplementation with SBR compared to 8 h concentrations at week 0 (*P* = 0.022). Estradiol-17β post-acetaminophen dose time-profile after the 4-week supplementation period was significantly lower than at week 0 across all time points (0 h (*P* = 0.005), 2 h (*P* = 0.007), 4 h (P = 0.040), 5 h (P = 0.021) and 6 h (*P* = 0.046)). Supplementation with SBR for 28 days was found to significantly lower estradiol-17β C_max_ (*P* = 0.004)_,_ and AUC_0-8h_ (*P* = 0.002) (Table 4).

**Table 4:** **Serum hormones profile of acetaminophen metabolites at baseline and 4 weeks**

Serum hormones	Week 0	Week 4	Difference from week 0 to week 4	***P***Value ^a^
**Total testosterone**				
C_max_(pmol/L)	17.38 ± 5.57	17.14 ± 4.57	-0.24 ± 3.22	0.906
T_max_(h)	0.85 ± 2.53	0.84 ± 2.53	0.00 ± 0.00	0.331
AUC_(0–8 hour)_ (pmol.h/L)	101.64 ± 36.38	99.77 ± 29.96	-1.87 ± 21.42	0.998
**Free testosterone**				
C_max_(pmol/L)	44.14 ± 13.33	44.38 ± 14.04	0.24 ± 5.30	0.924
T_max_(h)	0.85 ± 2.53	0.63 ± 2.01	-0.21 ± 2.11	0.667
AUC_(0–8 hour)_ (pmol.h/L)	245.71 ± 71.67	254.31 ± 64.02	8.60 ± 32.14	0.228
**Estradiol-17β**				
C_max_(pmol/L)	97.74 ± 25.87	84.32 ± 21.05	-13.42 ± 17.08	0.004
T_max_(h)	1.69 ± 2.53	1.90 ± 3.03	0.21 ± 3.89	0.817
AUC_(0–8 hour)_ (pmol.h/L)	687.08 ± 173.03	617.43 ± 120.00	-69.65 ± 88.00	0.002

C_max_, T_max_ and AUC_0-8h_ of serum hormones following administration of 1000 mg acetaminophen to participants at week 0 and after 4 weeks of SBR supplementation.

Values are presented as Mean ± SD^a^ Between group comparisons were made using *t*-test. Statistical comparisons for C_max_ and AUC_(0–8 hours)_ were conducted using log-transformed data.

C_max_ = maximum concentration; T_max_ = time of maximum concentration; AUC = area under the serum concentration time curve.

### Other anthropometric, biochemical and clinical observations

There were no significant differences in weight, BMI, heart rate or blood pressure after 4 weeks of supplementation with SBR. Participants had statistically significant increases in mean corpuscular volume (*P* < 0.001) and eosinophils (*P* = 0.049) and statistically significant decreases in mean corpuscular hemoglobin concentration (*P* = 0.030) and potassium (*P* = 0.001) after 4 weeks of supplementation with SBR. All values for these markers remained within acceptable laboratory reference ranges and were not of clinical significance.

**Figure 5:**
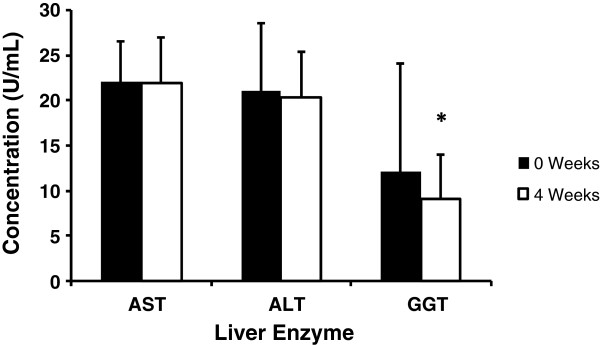
**The serum concentration of liver enzyme at baseline and 4 weeks.** Serum concentration of aspartate transaminase (AST), alanine transaminase (ALT) and γ-glutamyl transferase (GGT) at baseline (0 weeks) and following 4 weeks of SBR supplementation. Although no changes in AST and ALT were seen, a significant reduction in GGT was seen after 4 weeks of supplementation (*P = 0.002).

Liver function markers decreased from week 0 to week 4 after supplementation with SBR. Only GGT was statistically significant (*P* = 0.002), however this decrease remained within the clinically acceptable range (Figure 5).

Seven subjects reported a total of 14 adverse events during the study. One adverse event, indigestion, mild in severity, was categorized by the Principal Investigator as possibly related to the study supplement, SBR. There were no serious adverse events reported during the study.

## Discussion

This study provides evidence in humans that a supplement containing Spanish black radish positively influenced the detoxification of a 1000 mg dose of acetaminophen, suggesting an up-regulation of both phase I and phase II enzymes, which may provide hepatic cell protection. There are strong epidemiological and mechanistic data linking cruciferous vegetables with health outcomes such as decreased risk of various cancers [17]. SBR belongs to the Cruciferae family and contains high concentrations of glucosinolates, especially glucoraphasatin which represents over 65 percent of the total glucosinolates present [8]. The glucosinolate content, and more importantly, glucosinolate metabolites are thought to be major contributors to the health benefits associated with consuming cruciferous vegetables. High concentrations of glucosinolates are linked with a number of distinct cancer chemopreventive mechanisms, including the induction of antioxidant and detoxification genes through the activation of nuclear factor erythroid 2-related factor 2 and aryl hydrocarbon receptor, inhibition of pro-inflammatory reactions by repression of nuclear factor-kappa B, inhibition of CYP enzyme activity, inhibition of histone deacetylase, and stimulation of cell cycle arrest and apoptosis [18].

With SBR, glucoraphasatin is metabolized into rasphasatin [10]. An *in vitro* study of human hepatoma HepG2 cells has shown that raphasatin induces RNA expression of several phase I and phase II enzymes [8]. While this provides some evidence regarding the health benefits of SBR on hepatic function, further investigation is still required.

It is important to up-regulate both phase I and phase II detoxification enzymes because both phase I and phase II detoxification enzymes are important for regulating the concentration of toxic compounds in the body. Phase I enzymes produce pro-carcinogenic reactive intermediates [19, 20] and phase II detoxification enzymes also produce toxic compounds [21, 22]. The roles of the phase I and phase II enzymes are compound-dependent and therefore interdependent for effective detoxification of toxic compounds [23]. In addition, while some chemicals become active carcinogens after biotransformation by certain phase I enzymes such as the CYP family [17], isothiocyanates have been found to increase the expression of antioxidant and detoxification proteins such as glutathione S-transferase without increasing aryl hydrocarbon hydroxylase activity [18]. The current study suggests that both phase I and phase II enzymes were activated by the SBR supplement. Subjects on the SBR supplement had increased urinary mercapturate metabolite along with decreased estradiol-17β, which suggests that there was an increase in phase I metabolism. Subjects on SBR also had decreased plasma glucuronide and sulfate metabolites and an increase in urinary sulfate metabolite, suggesting an alteration of phase II metabolism. In addition, the decrease of unchanged acetaminophen in plasma and its increase in urine shows that SBR enhanced the clearance of unchanged acetaminophen.

A few human *Brassica* feeding studies have also reported an up-regulation in phase I [24–26] and phase II enzymes [26–29]. Mice supplemented with phenylbutyl isoselenocyanate, a derivative of isothiocyanate that is present in cruciferous vegetables as a glucosinolate precursor [30–32] showed a transient increase in expression of two phase I genes while phase II genes remained upregulated [33]. The animal studies suggest that the negative effects of phase I CYP activity may be overcome by phase II enzyme activity, thus demonstrating the interdependence between phase I and phase II detoxification enzymes.

Rodent models to evaluate the protective effects of pharmaceutical and nutraceutical interventions on acetaminophen-induced liver injury are widely used and accepted as clinically relevant in determining the mechanism of action of products [34–36]. The mouse model is considered to have close similarities to mechanisms of acetaminophen toxicity in human hepatocytes and to the mechanisms that occur in human overdose patients [36]. Therefore, we have extended this model and assessed the effect of SBR supplementation on human phase I and II metabolism of acetaminophen in the liver, as has previously been done for garlic extract [34]. Phase II assessment methods may be used to measure the clearance of acetaminophen which is metabolized through three phase II pathways: GSH conjugation, sulfation and glucuronidation. These pathways form urinary acetaminophen mercapturates, acetaminophen sulfate and acetaminophen glucuronide, respectively [7]. Low clearance of these acetaminophen metabolites is an indication that phase II reactions are impaired, and the measurement of these metabolites is a non-invasive method to determine the health of the liver.

The baseline profile of acetaminophen metabolites in this study was consistent with previous studies [37–39]. After 4 weeks of supplementation, urine acetaminophen glucuronide, acetaminophen sulfate, mercapturate and unchanged acetaminophen increased by 1%, 11%, 37% and 18%, respectively. Plasma acetaminophen glucuronide, acetaminophen sulfate, and unchanged acetaminophen decreased by 9%, 10%, and 40%, respectively. Decreased acetaminophen sulfate and unchanged acetaminophen in plasma corresponded to an increase in the urine, suggesting that subjects had more efficient metabolism and clearance of acetaminophen.

The serum total and free testosterone profiles post-acetaminophen dose after 4 weeks of supplementation were similar to baseline, but while post-acetaminophen dose serum estradiol-17β profiles followed similar patterns to baseline, the concentrations of the serum estradiol-17β at all time points, at maximum concentration, and over the course of absorption were significantly lower. Given that the more widely studied glucosinolate metabolite, indole-3-carbinol induces liver CYP1A1, 1A2 and 2B1/2 expression and increases estradiol-17β metabolism [40, 41], and a 20% freeze-dried radish diet stimulates CYP 1A1 and CYP 1A2 expression [10], the 16% decrease in fasting serum estradiol-17β concentrations suggests that phase I enzymes were induced by SBR from baseline to week 4 providing evidence that the glucosinolate metabolites and other potential actives remained functional despite the manufacturing process. The changes in plasma GGT and estradiol-17β levels and changes in acetaminophen metabolism with 4 weeks of SBR supplementation support the hypothesis that SBR may have a protective liver function.

### Limitations

As this was an open label study, the absence of a placebo group for comparison was certainly a limitation. Thus, a randomized, placebo-controlled clinical trial is clearly needed to establish the role of SBR in hepatic function. As well, the bioavailability profiles were measured for only 8 h. Measurement of the profiles over 24 h may have allowed for full documentation of acetaminophen clearance and established the time for plasma values to return to baseline. Furthermore, inclusion of both males and females would have provided information on gender specific effects of SBR.

## Conclusions

This study demonstrated that the supplement containing SBR positively altered the metabolism of acetaminophen, suggesting an effective increase in liver detoxification capacity in humans in the same manner as previous reports with whole food crucifers. Despite the large body of existing literature supporting the health benefits of cruciferous vegetables [17] and efforts to promote their consumption along with other fruits and vegetables, intake of these healthy foods has not increased [42]. The intake of cruciferous food supplements could provide an important alternative route to attain the same health benefits.

## Abbreviations

ALT: Alanine aminotransferase
AST: Aspartate aminotransferase
AUC: Concentration time curve
BMI: Body mass index
AUC: Area under the concentration time curve
C_max_: Maximum concentration
CYP: Cytochrome P450
GGT: Glutamyl transferase
GSH: Glutathione
HPLC: High performance liquid chromatography
NAPQI: N-acetyl-p-benzoquinone
SBR: Spanish black radish
T_max_: Time of maximum concentration.
